# Distinct Phenotypes of Non-Citizen Kidney Transplant Recipients in the United States by Machine Learning Consensus Clustering

**DOI:** 10.3390/medicines10040025

**Published:** 2023-03-27

**Authors:** Charat Thongprayoon, Pradeep Vaitla, Caroline C. Jadlowiec, Napat Leeaphorn, Shennen A. Mao, Michael A. Mao, Fahad Qureshi, Wisit Kaewput, Fawad Qureshi, Supawit Tangpanithandee, Pajaree Krisanapan, Pattharawin Pattharanitima, Prakrati C. Acharya, Pitchaphon Nissaisorakarn, Matthew Cooper, Wisit Cheungpasitporn

**Affiliations:** 1 Division of Nephrology and Hypertension, Department of Medicine, Mayo Clinic, Rochester, MN 55905, USAsupawit_d@hotmail.com (S.T.); pajaree_fai@hotmail.com (P.K.); 2 Division of Nephrology, University of Mississippi Medical Center, Jackson, MS 39216, USA; 3 Division of Transplant Surgery, Mayo Clinic, Phoenix, AZ 85054, USA; 4 Renal Transplant Program, University of Missouri-Kansas City School of Medicine/Saint Luke’s Health System, Kansas City, MO 64108, USA; 5 Division of Transplant Surgery, Mayo Clinic, Jacksonville, FL 32224, USA; 6 Division of Nephrology and Hypertension, Department of Medicine, Mayo Clinic, Jacksonville, FL 32224, USA; 7 School of Medicine, University of Missouri-Kansas City, Kansas City, MO 64108, USA; 8 Department of Military and Community Medicine, Phramongkutklao College of Medicine, Bangkok 10400, Thailand; 9 Division of Nephrology, Department of Internal Medicine, Faculty of Medicine Thammasat University, Pathum Thani 12120, Thailand; 10 Division of Nephrology, Texas Tech Health Sciences Center El Paso, El Paso, TX 79905, USA; 11 Department of Medicine, Division of Nephrology, Massachusetts General Hospital, Harvard Medical School, Boston, MA 02114, USA; 12 Medstar Georgetown Transplant Institute, Georgetown University School of Medicine, Washington, DC 21042, USA

**Keywords:** kidney transplant, transplantation, clustering, machine learning, non-U.S. citizen

## Abstract

Background: Better understanding of the different phenotypes/subgroups of non-U.S. citizen kidney transplant recipients may help the transplant community to identify strategies that improve outcomes among non-U.S. citizen kidney transplant recipients. This study aimed to cluster non-U.S. citizen kidney transplant recipients using an unsupervised machine learning approach; Methods: We conducted a consensus cluster analysis based on recipient-, donor-, and transplant- related characteristics in non-U.S. citizen kidney transplant recipients in the United States from 2010 to 2019 in the OPTN/UNOS database using recipient, donor, and transplant-related characteristics. Each cluster’s key characteristics were identified using the standardized mean difference. Post-transplant outcomes were compared among the clusters; Results: Consensus cluster analysis was performed in 11,300 non-U.S. citizen kidney transplant recipients and identified two distinct clusters best representing clinical characteristics. Cluster 1 patients were notable for young age, preemptive kidney transplant or dialysis duration of less than 1 year, working income, private insurance, non-hypertensive donors, and Hispanic living donors with a low number of HLA mismatch. In contrast, cluster 2 patients were characterized by non-ECD deceased donors with KDPI <85%. Consequently, cluster 1 patients had reduced cold ischemia time, lower proportion of machine-perfused kidneys, and lower incidence of delayed graft function after kidney transplant. Cluster 2 had higher 5-year death-censored graft failure (5.2% vs. 9.8%; *p* < 0.001), patient death (3.4% vs. 11.4%; *p* < 0.001), but similar one-year acute rejection (4.7% vs. 4.9%; *p* = 0.63), compared to cluster 1; Conclusions: Machine learning clustering approach successfully identified two clusters among non-U.S. citizen kidney transplant recipients with distinct phenotypes that were associated with different outcomes, including allograft loss and patient survival. These findings underscore the need for individualized care for non-U.S. citizen kidney transplant recipients.

## 1. Introduction

In the United States (U.S.), the National Organ Transplant Act (NOTA) and the United Network for Organ Sharing (UNOS)/Organ Procurement and Transplantation Network (OPTN) do not restrict access to organ transplant according to citizenship status. The only requirement is that medical criteria be utilized in organ allocation once a patient has been listed for transplant [1,2,3]. The “10%” rule (changed to “5% rule” in 1994) was perceived by many as a cap on the number of non-U.S. citizens able to be listed for transplant [3]. While this created an informal barrier to transplantation for non-U.S. citizens, there was no detailed audit as a result of this policy [3]. UNOS subsequently revised their policy in 2012 by disregarding thresholds, and they instead collected data on each candidate’s citizenship and residency status [3].

There are more than 6500 non-U.S. citizens residing in the U.S. with end-stage kidney disease (ESKD), and who are on maintenance dialysis [4]. A recent study demonstrated that 1.2% of all transplants performed in the U.S. were non-citizen/non-resident patients between 2013 and 2016, with 402 deceased donor kidney transplants performed for non-citizen/non-resident patients [2]. Studies have also suggested that the deceased donor kidney transplant waiting time for non-U.S. citizens does not differ from that of U.S. citizens, and non-U.S. citizens also receive comparable quality of decease donor kidney to U.S. citizens [2,5]. Thus, among non-U.S. citizen ESKD patients with Medicaid (a U.S. public insurance program) and access to immunosuppressive drugs, studies have demonstrated comparable outcomes to those of U.S. citizens [4,6]. Nevertheless, non-U.S. citizens are a unique patient population with important factors that may impact medical outcomes, such as social, educational, immunological, and donor factors, that have not been well studied [2,4,7,8,9,10].

Artificial intelligence and machine learning (ML) have been utilized to aid clinical decision support tools in organ transplantation [11,12,13,14,15,16]. Unsupervised consensus clustering is a ML approach employed to identify distinct subtypes and novel data patterns [17,18,19]. It can find similarities and heterogeneities among diverse data variables and differentiate them into clinically useful clusters that may deliver new insight [17,18]. Recent studies have demonstrated that distinct subtypes identified by ML consensus clustering approach can forecast different clinical outcomes [20,21]. Given that non-citizen kidney transplant recipients are heterogeneous, a better understanding of the different phenotypes of non-citizen kidney transplant recipients may help the transplant community to identify strategies that improve outcomes among this patient population.

In this study, we analyzed the UNOS/OPTN database from 2010 through 2019, utilizing an unsupervised ML clustering algorithm to identify clusters of non-U.S. citizen kidney transplant recipients, and we then assessed the clinical outcomes among these distinct clusters.

## 2. Materials and Methods

### 2.1. Data Source

This study was conducted using the OPTN/UNOS database to identify adult kidney-only transplant recipients in the U.S. from 2010 to 2019. Patients with non-U.S. citizenship status were included. For patients with multiple kidney transplants during the study period, the first kidney transplant was selected for analysis. This study received approval from the Mayo Clinic Institutional Review Board (IRB number 21-007698).

### 2.2. Data Collection

The recipient, donor, and transplant-related variables in the UNOS/OPTN database were used in the ML cluster analysis, including recipient age, sex, race, body mass index (BMI), cause of end-stage kidney disease, dialysis duration, panel reactive antibody (PRA), kidney retransplant, comorbidities, hepatitis B, hepatitis C, human immunodeficiency virus (HIV) serostatus, ABO incompatibility, Karnofsky functional performance score, working income, insurance, U.S. residency status, education, and serum albumin, as well as kidney donor type, donor age, sex, and race, history of hypertension in donor, HLA mismatch, kidney donor profile index (KDPI), kidney on pump, cold ischemia time, delay graft function, allocation type, Cytomegalovirus (CMV) and Epstein–Barr virus (EBV) status, and induction and maintenance immunosuppression. The U.S. residency status was categorized into four groups: (1) non-U.S. citizen/U.S. resident, (2) resident alien, (3) non-resident alien, (4) non-U.S. citizen/non-U.S. resident that travelled to the U.S. for transplant, and (5) non-U.S. citizen/non-U.S. resident that travelled to the U.S. for reasons other than transplant. Resident aliens were defined as citizens who were from another country and who lived in the U.S. and/or had resident status by law or visa. Non-resident alien is a person who was not a U.S. citizen and who did not meet either the “green card” test or the “substantial presence” test [7]. All extracted variables had missing data < 5% (Table S1). Missing data were imputed using the multivariable imputation by chained equation (MICE) approach [22].

### 2.3. Clustering Analysis

Unsupervised ML was applied by conducting a consensus clustering approach to categorize clinical phenotypes of non-U.S. citizen kidney transplant recipients [23]. A pre-specified subsampling parameter of 80% with 100 iterations with the number of potential clusters (k) ranging from 2 to 10 was utilized to avoid constructing an extreme number of clusters that would not be clinically meaningful. The optimal number of clusters was chosen by analyzing the consensus matrix (CM) heat map, cumulative distribution function (CDF), cluster-consensus plots with the within-cluster consensus scores, and the proportion of ambiguously clustered (PAC) pairs. The within-cluster consensus score, ranging from 0 to 1, was represented as the mean consensus value for all pairs of individuals belonging to the same group [17]. A value closer to one implies more satisfactory cluster stability. The PAC pairs, ranging from 0 to 1, were computed as the proportion of all sample pairs with consensus values falling within the predetermined boundaries [24]. The detailed consensus cluster algorithms used in this study for reproducibility are provided in the Online Supplementary Materials.

### 2.4. Outcomes

Post-transplant outcomes consisted of death-censored graft failure, patient death within five years after transplantation, and acute allograft rejection within one year of transplantation. We defined death-censored graft failure as the need for dialysis or kidney retransplant, with patients censored based on death or who at last follow-up date reported to the UNOS/OPTN database.

### 2.5. Statistical Analysis

Statistical analyses were performed to characterize the differences among the clusters to which non-U.S. citizen kidney transplant patients were assigned via the consensus clustering approach. The differences in clinical characteristics between the assigned clusters were tested using an analysis of variance test or a Kruskal–Wallis test, as appropriate, for the continuous variables, and a Chi-squared test for the categorical variables. The key characteristics of each cluster were determined using the standardized mean difference between each cluster and the overall cohort, with a cut-off of >0.3.

The differences in post-transplant outcomes, including death-censored graft failure, patient death within five years of kidney transplant, and allograft rejection within one year of kidney transplant, were evaluated among the assigned clusters. The hazard ratios (HRs) for death-censored graft failure and patient death based on the assigned clusters were obtained using the Cox proportional hazard analysis. Since the OPTN/UNOS database did not specify the date of allograft rejection occurrence, the odds ratio for one-year allograft rejection was obtained using a logistic regression analysis for each of the assigned clusters. A multivariable analysis was not performed to adjust for differences in clinical characteristics among the assigned clusters because an unsupervised ML consensus clustering approach utilizes these characteristics to classify clusters.

All analyses were conducted using R, version 4.0.3 (RStudio, Inc., Boston, MA, USA; http://www.rstudio.com/, accessed on 21 July 2022); ConsensusClusterPlus package (version 1.46.0) for the consensus clustering analysis, and the MICE command in R for multivariable imputation by chained equation [22].

## 3. Results

There were 158,367 kidney transplant recipients from 2010 to 2019 in the U.S. Of these, 11,300 (7%) had non-U.S. citizenship status. Consensus clustering analysis was thus performed in 11,300 non-U.S. citizen kidney transplant recipients. Fifty-five percent were non-U.S. citizen/U.S. resident, 29% were resident alien, 5% were non-resident alien, 3% were non-U.S. citizen/non-U.S. resident who travelled to the U.S. for transplant, and 9% were non-U.S. citizen/non-U.S. resident who travelled to the U.S. for reasons other than transplant. Table S2 shows the country of citizenship of the non-U.S. citizens/non-U.S. residents.

**Figure 1:**
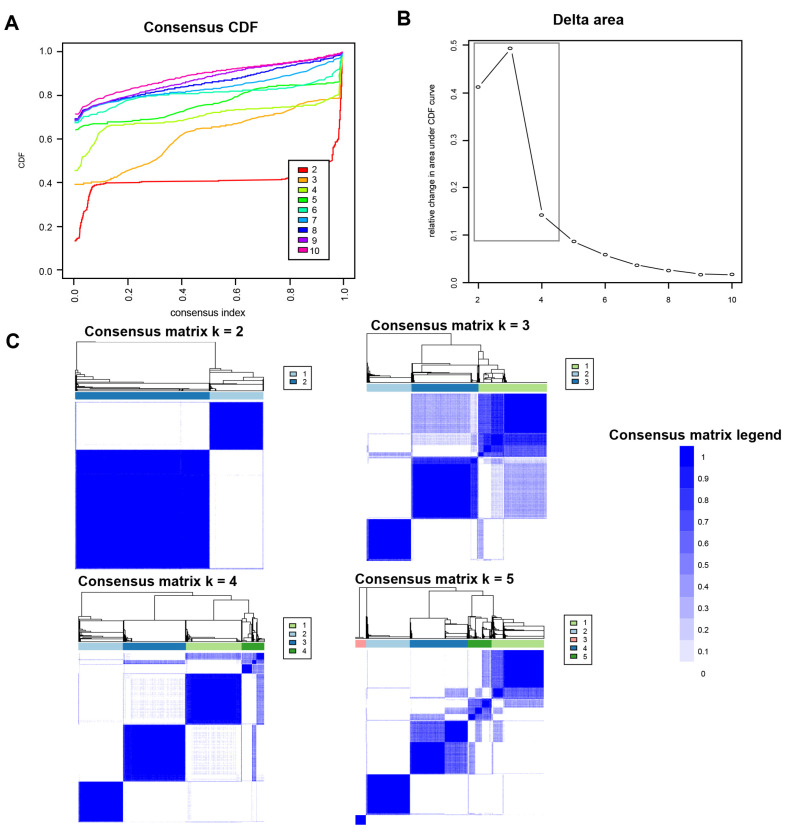
(**A**) CDF plot displaying consensus distributions for each k; (**B**) delta area plot reflecting the relative changes in the area under the CDF curve; (**C**) consensus matrix heat map depicting consensus values on a white to blue color scale of each cluster.

**Figure 2:**
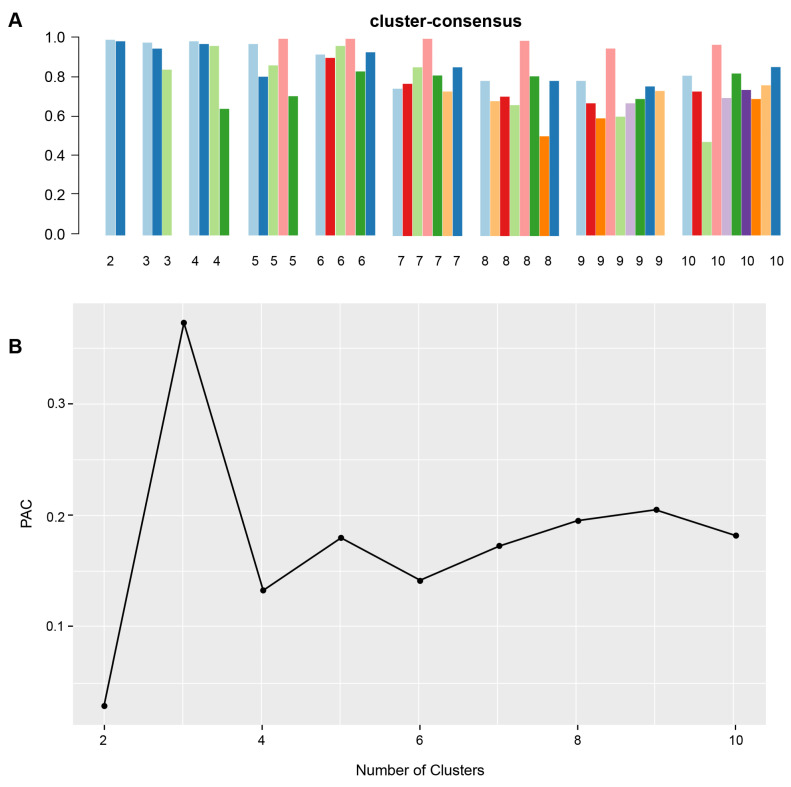
(**A**) The bar plot represents the mean consensus score for different numbers of clusters (K ranges from two to ten). Different colors indicate different cluster groups.; (**B**) the PAC values assess ambiguously clustered pairs.

Figure 1A shows the CDF plot consensus distributions for each identified cluster of non-U.S. citizen kidney transplant recipients; the delta area plot shows the relative change in area under the CDF curve (Figure 1B). The largest changes in area emerged between k = 2 and k = 4, at which point the relative increase in area became noticeably smaller. As shown in the CM heat map (Figure 1C, Supplementary Figures S1–S9), the ML algorithm identified cluster 2 with clear boundaries, indicating good cluster stability over repeated iterations. The mean cluster consensus score was highest in cluster 2 (Figure 2A). In addition, favorable low PAC pairs were demonstrated for two clusters (Figure 2B). Thus, using baseline characteristics at the time of transplantation, the consensus clustering analysis identified two clusters that best represented the data pattern of our non-U.S. citizen kidney transplant recipients.

### 3.1. Clinical Characteristics of Each Non-U.S. Citizen Kidney Transplant Cluster

**Figure 3:**
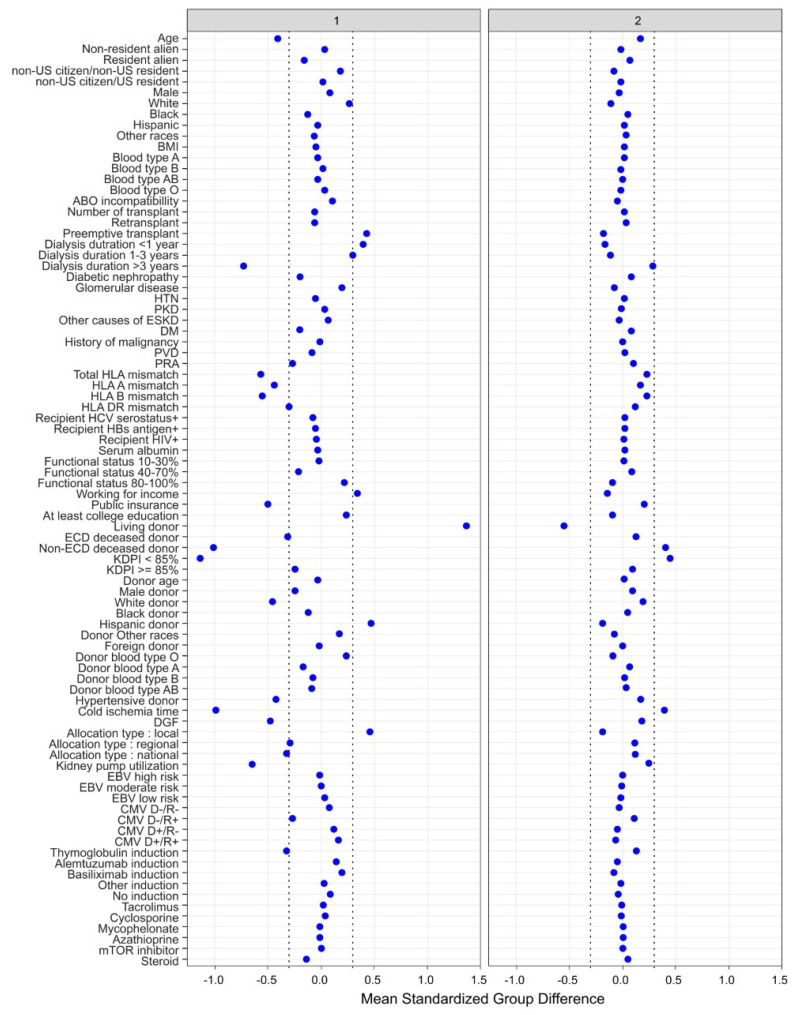
The standardized differences across two clusters for each of the baseline parameters. The x axis is the standardized differences value, and the y axis shows baseline parameters. The dashed vertical lines represent the standardized differences cutoffs of <−0.3 or >0.3. Abbreviations: BMI: body mass index, CMV: cytomegalovirus, D: donor, DGF: delayed graft function, DM: diabetes mellitus, EBV: Epstein–Barr virus, ECD: extended criteria donor, ESKD: end stage kidney disease, GN: glomerulonephritis, HBs: hepatitis B surface, HCV: hepatitis C virus, HIV: human immunodeficiency virus, HLA: human leucocyte antigen, HTN: hypertension, KDPI: kidney donor profile index, mTOR: mammalian target of rapamycin, PKD: polycystic kidney disease, PRA: panel reactive antibody, PVD: peripheral vascular disease, R: recipient.

There were two distinct clinical clusters identified using a ML consensus clustering analysis. Cluster 1 had 3226 (29%) patients and cluster 2 (71%) had 8074 patients. These two identified clusters had distinct clinical characteristics, as shown in Table 1. The key characteristics of cluster 1 patients included young age, preemptive kidney transplant or dialysis duration of less than 1 year, working income, private insurance, non-hypertensive donors, and Hispanic living donor with low number of HLA mismatch. Consequently, cluster 1 patients had less cold ischemia time, a lower proportion of machine-perfused kidneys, and a lower incidence of delayed graft function after kidney transplant compared to cluster 2. In contrast, the key characteristics of cluster 2 patients included having non-ECD deceased donors with a KDPI < 85% (Figure 3).

Supplementary Figure S10 and Table S3 show the proportion of cluster 1 and cluster 2 patients based on the UNOS regions. Overall, kidney transplantation for non-U.S. citizens in the United States from 2010 to 2019 occurred most often in Region 5 (Arizona, California, Nevada, New Mexico, and Utah), followed by Region 9 (New York and Western Vermont) and Region 4 (Oklahoma and Texas) (Table S2). Region 7 (Illinois, Minnesota, North Dakota, South Dakota, and Wisconsin) had the highest proportion of cluster 1 (42%), whereas region 11 (Kentucky, North Carolina, South Carolina, Tennessee, and Virginia) had the highest proportion of cluster 2 (81%).

### 3.2. Post-Transplant Outcomes of Each Non-U.S. Citizen Kidney Transplant Cluster

**Figure 4:**
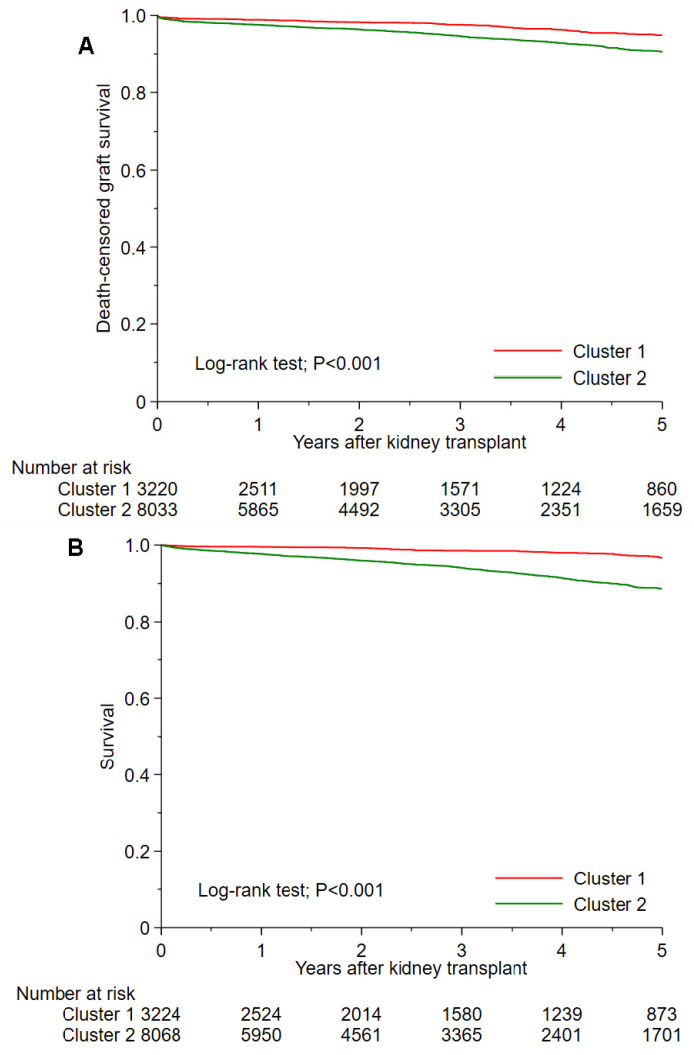
(**A**) Death-censored graft survival and (**B**) patient survival after kidney transplant among two unique clusters of non-citizen kidney transplant recipients in the U.S.

Table 2 shows post-transplant outcomes based on cluster. The 1-year and 5-year death-censored graft failure was 1.2% and 5.2% in cluster 1, and 2.7% and 9.8% in cluster 2, respectively (*p* < 0.001) (Figure 4A). Cluster 2 had higher 1-year and 5-year death-censored graft failure than cluster 1 with a HR of 2.22 (95% CI 1.59–3.19) and 2.02 (95% CI 1.63–2.52), respectively. The 1-year and 5-year death was 0.5% and 3.4% in cluster 1, and 2.4% and 11.4% in cluster 2, respectively (*p* < 0.001) (Figure 4B). Cluster 2 had higher 1-year and 5-year death than cluster 1 with HRs of 4.68 (95% CI 2.86–8.27) and 3.92 (95% CI 2.98–5.17), respectively. The incidence of 1-year acute allograft rejection was comparable between cluster 1 and cluster 2 (4.7% vs. 4.9%; *p* = 0.63).

## 4. Discussion

Our unsupervised ML approach identified two clinically distinct clusters of non-U.S. citizen kidney transplant recipients with differing post-transplant outcomes. Cluster 1 patients, accounting for 28.5% of non-U.S. citizen kidney transplant recipients, were featured by young age recipients receiving preemptive kidney transplant or a short dialysis duration of less than one year. Patients in cluster 1 had working incomes and private insurance. Most donors in cluster 1 were non-hypertensive Hispanic living donors with a low number of HLA mismatches. In contrast, cluster 2 patients, accounting for 71.5% of non-U.S. citizen kidney transplant recipients, were primarily characterized by receipt of non-ECD deceased donor with a KDPI < 85% (89.5% had KDPI < 85%, 9.6% had KDPI ≥ 85%, and only 1.0% had living donors). Kidney transplants in cluster 2 patients had a longer cold ischemia time, higher utilization of machine-perfusion, and a higher incidence of DGF. While cluster 2 patients had a higher degree of HLA mismatches and DGF than cluster 1, they received more thymoglobulin and less steroid-free regimens compared to cluster 1. This may have resulted in the comparable 1-year acute rejection rate between the two groups. Nevertheless, cluster 2 patients had significantly higher 5-year death-censored graft failure and mortality.

A previous study using the U.S. Renal Data was conducted to assess outcomes of adult kidney transplant recipients (years 1990 and 2011) with Medicaid [4]. The investigators demonstrated that kidney transplant recipients, regardless of U.S. citizenship status, had comparable outcomes [4]. These findings would thus be reassuring as to the safety of kidney transplants for non-U.S. citizens. The previous study was limited, however, as it only addressed individuals with public insurance, thus ruling out the majority of non-U.S. citizen recipients in Cluster 1 of our study. Our study provides novel understanding of the phenotypes of non-U.S. citizens regardless of insurance or socioeconomic status.

It is notable that UNOS/OPTN data on non-U.S. citizenship candidate listings were recorded as “non-U.S. citizen/U.S. resident” or “non-U.S. citizen/non-U.S. resident” after March 2012 [3]. Prior to March 2012, non-U.S. citizenship candidates on the waiting list were documented as “resident alien” or “nonresident alien,” and they were not reassigned to the updated status [3]. In our study, we did not exclude patients with “resident alien” or “nonresident alien” status in order to truly capture and represent all non-U.S. citizen kidney transplant recipients in the United States. While the subtype of U.S. citizenship status was not one of the key phenotypes that differentiated the two identified clusters, we found that cluster 2 patients has a higher proportion of resident aliens (also termed permanent resident or a lawful permanent resident) [4]. Non-citizen/non-resident status included both patients that traveled to the U.S. for either the purpose of seeking an organ transplant or reasons other than transplantation [4,10]. Non-citizen/non-resident patients could be foreign students or business people traveling to the U.S, consistent with the findings of recipients with higher education levels and higher working incomes with private insurance. Individuals falling into the non-citizen/non-resident status may also be those who traveled to the United States with a living donor. These socioeconomic factors have been shown to be associated with better graft and recipient outcomes [7]. During the study period, the top five reported countries of citizenship of non-U.S. citizen/non-U.S. resident transplant recipients who traveled to the U.S. for transplant were Kuwait, Qatar, Mexico, Saudi Arabia, and United Arab Emirates. The top five reported countries of citizenship of non-U.S. citizen/non-U.S. resident recipients who traveled to the U.S. for other reasons were Mexico, El Salvador, India, Guatemala, and Chile (Table S1). While our study demonstrated a higher proportion of non-citizen/non-resident patients that traveled to the U.S. for either the purposes of seeking an organ transplant or for reasons other than transplant in cluster 1, as compared to cluster 2, the overall number of non-U.S. citizen/ non-U.S. resident recipients who traveled to the U.S. for a reason other than transplantation was higher in cluster 2 (650 patients (8%)) than cluster 1 (344 patients (11%)).

Our study has several limitations. We used the UNOS database to assess the phenotypes of non-citizen adult kidney transplant recipients in the United States. Thus, the findings of our study are not representative of non-citizen kidney transplant recipients in other countries [25,26] or pediatric transplant recipients [10,27]. Second, while there have been concerns about kidney transplant outcomes among “undocumented” aliens or residents [3], there are no “illegal” or “undocumented” terms identified in the UNOS database to identify patients who did not have a visa or who had overstayed the duration of their visa. While undocumented immigrants are considered as non-resident aliens [4], the expression “non-resident alien” is broad and also includes individuals granted permission by the U.S. government to enter the U.S. on a temporary basis as a non-immigrant alien for purposes which include tourism, business, education, medical care, or temporary employment. While a number of non-resident aliens have higher education without economic barriers, some others may enter into medical care with few resources, lack of acculturation, minimal health insurance, and little understanding of strategies to navigate the complex healthcare system, all of which may delay access to needed care [2,8]. Given the heterogeneity of non-resident aliens, a ML approach may have unique advantages for identifying distinct phenotypes. As of April 2012, undocumented immigrants are considered as “non-U.S. citizen/U.S. resident” in the updated terminology [3,4,10]. However, the term “non-U.S. citizen/U.S. resident” is still not specific to undocumented immigrants, and this term also includes a permanent resident or a lawful permanent resident. We found a comparable proportion of non-U.S. citizen/U.S. resident status in both cluster 1 and cluster 2. Given no definite identification of undocumented immigrants in the database, future studies to identify phenotypes of these vulnerable groups of patients are needed. Furthermore, kidney transplant recipients undergo rigorous screening and must satisfy specific criteria as part of the selection process. Consequently, the non-U.S. citizen/U.S. resident transplantation population shown here may not reflect the general trends described in other non-U.S. citizen patient populations outside of transplantation [28,29,30].

## 5. Conclusions

Our ML clustering approach successfully identified two clusters among non-U.S. citizen kidney transplant recipients with distinct phenotypes that were associated with different outcomes, including allograft loss and patient survival. Furthermore, there are different distributions among the 11 geographic OPTN regions in our identified clusters, which may help identify future strategies for the improvement of outcomes for non-U.S. citizen kidney transplant recipients.

## Figures and Tables

**Table 1:** Clinical characteristics according to clusters of non-U.S. citizen kidney transplant recipients.

	All	Cluster 1	Cluster 2	*p*-Value
	(*n* = 11,300)	(*n* = 3226)	(*n* = 8074)	
Recipient Age (year)	48.4 ± 13.6	42.8 ± 14.0	50.6 ± 12.8	<0.001
Recipient male sex	7058 (62.5)	2144 (66.5)	4914 (60.9)	<0.001
Recipient race				
White	1117 (9.9)	569 (17.6)	548 (6.8)	<0.001
Black	1101 (9.7)	198 (6.1)	903 (11.2)	<0.001
Hispanic	7023 (62.2)	1958 (60.7)	5065 (62.7)	0.04
Other	2059 (18.2)	501 (15.5)	1558 (19.3)	<0.001
ABO blood group				0.01
A	3570 (31.6)	983 (30.5)	2587 (32.0)	
B	498 (4.4)	117 (3.6)	381 (4.7)	
AB	1594 (14.1)	467 (14.5)	1127 (14.0)	
O	5638 (49.9)	1659 (51.4)	3979 (49.3)	
Body mass index (kg/m^2^)	26.3 ± 4.8	26.1 ± 4.9	26.4 ± 4.7	<0.001
Kidney retransplant	618 (5.5)	130 (4.0)	488 (6.0)	<0.001
Dialysis duration				<0.001
Preemptive	1018 (9.0)	690 (21.4)	328 (4.1)	
<1 year	1106 (9.8)	692 (21.5)	414 (5.1)	
1–3 years	6881 (60.9)	805 (25.0)	6076 (75.3)	
>3 years	2295 (20.3)	1039 (32.2)	1256 (15.6)	
Cause of end-stage kidney disease				
Diabetes mellitus	3087 (27.3)	596 (18.5)	2491 (30.9)	<0.001
Hypertension	2473 (21.9)	961 (29.8)	1512 (18.7)	<0.001
Glomerular disease	3042 (26.9)	783 (24.3)	2259 (28.0)	<0.001
PKD	612 (5.4)	203 (6.3)	409 (5.1)	0.009
Other	2086 (18.5)	683 (21.2)	1403 (17.4)	<0.001
Comorbidity				
Diabetes mellitus	3588 (31.8)	714 (22.1)	2874 (35.6)	<0.001
Malignancy	326 (2.9)	82 (2.5)	244 (3.0)	0.17
Peripheral vascular disease	788 (7.0)	156 (4.8)	632 (7.8)	<0.001
PRA	0 (0–23)	0 (0–0)	0 (0–34)	<0.001
Positive HCV serostatus	292 (2.6)	46 (1.4)	246 (3.0)	<0.001
Positive HBs antigen	261 (2.3)	50 (1.5)	211 (2.6)	0.001
Positive HIV serostatus	62 (0.5)	7 (0.2)	55 (0.7)	0.003
Functional status				<0.001
10–30%	19 (0.2)	2 (0.1)	17 (0.2)	
40–70%	4501 (39.8)	943 (29.2)	3558 (44.1)	
80–100%	6780 (60.0)	2281 (70.7)	4499 (55.7)	
Working income	3193 (28.3)	1407 (43.6)	1786 (22.1)	<0.001
Public insurance	8052 (71.3)	1557 (48.3)	6495 (80.4)	<0.001
U.S. residency status				<0.001
Non-U.S. citizen/U.S. resident	6217 (55.0)	1807 (56.0)	4410 (54.6)	
Non-U.S. citizen/non-U.S. resident, travel to U.S. for transplant	296 (3)	215 (7)	81 (1)	
Non-U.S. citizen/non-U.S. resident, travel to U.S. for reason other than transplant				
Resident alien	994 (9)	344 (11)	650 (8)	
Non-resident alien				
	3288 (29.1)	696 (21.6)	2592 (32.1)	
	505 (4.5)	164 (5.1)	341 (4.2)	
Undergraduate education or above	3345 (29.6)	1300 (40.3)	2045 (25.3)	<0.001
Serum albumin (g/dL)	4.1 ± 0.6	4.0 ± 0.6	4.1 ± 0.6	0.01
Kidney donor status				<0.001
Non-ECD deceased	7196 (63.7)	470 (14.6)	6726 (83.3)	
ECD deceased	1307 (11.6)	38 (1.2)	1269 (15.7)	
Living	2797 (24.8)	2718 (84.3)	79 (1.0)	
ABO incompatibility	21 (0.2)	21 (0.7)	0 (0.0)	<0.001
Donor age	38.3 ± 15.6	37.8 ± 12.4	38.5 ± 16.7	0.01
Donor male sex	6377 (56.4)	1425 (44.2)	4952 (61.3)	<0.001
Donor race				
White	5629 (49.8)	864 (26.8)	4765 (59.0)	<0.001
Black	1117 (9.9)	200 (6.2)	917 (11.4)	<0.001
Hispanic	3623 (32.1)	1742 (54.0)	1881 (23.3)	<0.001
Other	931 (8.2)	420 (13.0)	511 (6.3)	<0.001
History of hypertension in donor	2358 (20.9)	104 (3.2)	2254 (27.9)	<0.001
KDPI				<0.001
Living donor	2797 (24.8)	2718 (84.3)	79 (1.0)	
KDPI < 85	7708 (68.2)	485 (15.0)	7223 (89.5)	
KDPI ≥ 85	795 (7.0)	23 (0.7)	772 (9.6)	
HLA mismatch				
A	1 (1–2)	1 (0–1)	2 (1–2)	<0.001
B	2 (1–2)	1 (1–2)	2 (1–2)	<0.001
DR	1 (1–2)	1 (0–1)	1 (1–2)	<0.001
ABDR	4 (3–5)	3 (2–4)	5 (4–5)	<0.001
Cold ischemia time (hours)	14.5 ± 11.0	3.6 ± 4.9	18.9 ± 9.7	<0.001
Kidney on pump	3715 (32.9)	62 (1.9)	3653 (45.2)	<0.001
Delay graft function	2844 (25.2)	152 (4.7)	2692 (33.3)	<0.001
Allocation type				<0.001
Local	8905 (78.8)	3151 (97.7)	5754 (71.3)	
Regional	1049 (9.3)	27 (0.8)	1022 (12.7)	
National	1344 (11.9)	48 (1.5)	1296 (16.1)	
Foreign	2 (0.0)	0 (0.0)	2 (0.0)	
EBV status				
Low risk	63 (0.6)	25 (0.8)	38 (0.5)	0.05
Moderate risk	10,341 (91.5)	2959 (91.7)	7382 (91.4)	0.61
High risk	896 (7.9)	242 (7.5)	654 (8.1)	0.29
CMV status				
D-/R-	506 (4.5)	199 (6.2)	307 (3.8)	<0.001
D-/R+	3264 (28.9)	528 (16.4)	2736 (33.9)	<0.001
D+/R+	6819 (60.3)	2201 (68.2)	4618 (57.2)	<0.001
D+/R-	711 (6.3)	298 (9.2)	413 (5.1)	<0.001
Induction immunosuppression				
Thymoglobulin	6822 (60.4)	1440 (44.6)	5382 (66.7)	<0.001
Alemtuzumab	1438 (12.7)	557 (17.3)	881 (10.9)	<0.001
Basiliximab	2679 (23.7)	1033 (32.0)	1646 (20.4)	<0.001
Other	144 (1.3)	53 (1.6)	91 (1.1)	0.03
No induction	819 (7.2)	305 (9.5)	514 (6.4)	<0.001
Maintenance Immunosuppression				
Tacrolimus	10,589 (93.7)	3036 (94.1)	7553 (93.5)	0.27
Cyclosporine	159 (1.4)	56 (1.7)	103 (1.3)	0.06
Mycophenolate	10,730 (95.0)	3052 (94.6)	7678 (95.1)	0.28
Azathioprine	21 (0.2)	3 (0.1)	18 (0.2)	0.15
mTOR inhibitors	51 (0.5)	15 (0.5)	36 (0.4)	0.89
Steroid	7942 (70.3)	2065 (64.0)	5877 (72.8)	<0.001

Abbreviations: BMI: body mass index, CMV: cytomegalovirus, D: donor, EBV: Epstein–Barr virus, ECD: extended criteria donor, HBs: hepatitis B surface, HCV: hepatitis C virus, HIV: human immunodeficiency virus, KDPI: kidney donor profile index, mTOR: mammalian target of rapamycin, PKD: polycystic kidney disease, PRA: panel reactive antibody, R: recipient. SI conversion: serum albumin: g/dL × 10 = g/L.

**Table 2:** Post-transplant outcomes according to the clusters.

	Cluster 1	Cluster 2
One-year death-censored graft failure	1.2%	2.7%
HR for 1-year death-censored graft failure	1 (ref)	2.22 (1.59–3.19)
Five-year death-censored graft failure	5.2%	9.8%
HR for 5-year death-censored graft failure	1 (ref)	2.02 (1.63–2.52)
One-year death	0.5%	2.4%
HR for 1-year death	1 (ref)	4.68 (2.86–8.27)
Five-year death	3.4%	11.4%
HR for 5-year death	1 (ref)	3.92 (2.98–5.17)
One-year acute rejection	4.7%	4.9%
OR for 1-year acute rejection	1 (ref)	1.05 (0.87–1.27)

## Data Availability

Data will be made available by the authors upon reasonable request.

## Supplementary Materials

The following supporting information can be downloaded at: https://www.mdpi.com/article/10.3390/medicines10040025/s1, References [17,22,23,24,31,32,33,34,35,36] are cited in the Supplementary Materials. Figure S1. Consensus matrix heat map (k = 2) depicting consensus values on a white to blue color scale of each cluster; Figure S2. Consensus matrix heat map (k = 3) depicting consensus values on a white to blue color scale of each cluster; Figure S3. Consensus matrix heat map (k = 4) depicting consensus values on a white to blue color scale of each cluster; Figure S4. Consensus matrix heat map (k = 5) depicting con-sensus values on a white to blue color scale of each cluster; Figure S5. Consensus matrix heat map (k = 6) depicting consensus values on a white to blue color scale of each cluster; Figure S6. Consensus matrix heat map (k = 7) depicting consensus values on a white to blue color scale of each cluster; Figure S7. Consensus matrix heat map (k = 8) depicting consensus values on a white to blue color scale of each cluster; Figure S8. Consensus matrix heat map (k = 9) depicting con-sensus values on a white to blue color scale of each cluster; Figure S9. Consensus matrix heat map (k = 10) depicting consensus values on a white to blue color scale of each cluster. Figure S10. A. Proportion of clusters according to the regions. B. OPTN regions; Table S1. The number and percentages of missing data; Table S2. Proportion of clusters according to the regions.
